# Non-HACEK gram-negative bacilli infective endocarditis: data from a retrospective German cohort study

**DOI:** 10.1007/s15010-024-02392-w

**Published:** 2024-09-19

**Authors:** Juliane Dörfler, Herko Grubitzsch, Matthias Schneider-Reigbert, Miralem Pasic, Frieder Pfäfflin, Miriam Stegemann, Leif E. Sander, Florian Kurth, Tilman Lingscheid

**Affiliations:** 1 Department of Infectious Diseases, Respiratory Medicine and Critical Care, Charité – Universitätsmedizin Berlin, Corporate Member of Freie Universität Berlin and Humboldt-Universität zu Berlin, Augustenburger Platz 1, 13353 Berlin, Germany; 2 Deutsches Herzzentrum der Charité - Klinik für Herz-, Thorax- und Gefäßchirurgie, (Department of Cardiothoracic and Vascular Surgery), Augustenburger Platz 1, 13353 Berlin, Germany; 3 Deutsches Herzzentrum der Charité - Klinik für Kardiologie, Angiologie und Intensivmedizin (Department of Cardiology, Angiology and Intensive Care Medicine), Augustenburger Platz 1, 13353 Berlin, Germany; 4 Charité – Universitätsmedizin Berlin, Corporate Member of Freie Universität Berlin and Humboldt-Universität zu Berlin, Charitéplatz 1, 10117 Berlin, Germany; 5 DZHK (German Center for Cardiovascular Research) Partner Site Berlin, Berlin, Germany; 6 German Center for Lung Research, DZL, Berlin, Germany; 7 Berlin Institute of Health, Berlin, Germany

**Keywords:** Enterobacterales, Combination antibiotic therapy, Cardiac surgery, Echocardiography, Endocarditis team, Endoplastitis

## Abstract

**Purpose:**

Infective endocarditis caused by *non-HACEK* gram-negative bacilli (GNB-IE) is rare but associated with significant morbidity and case fatality. Evidence on optimal treatment and management is limited. We aimed to describe the characteristics and management of GNB-IE patients, investigating factors associated with disease acquisition and unfavorable outcomes.

**Methods:**

We conducted a retrospective descriptive single-center study (tertiary care and referral hospital) between 2015 and 2021, including adult patients with definite GNB-IE. We reviewed demographic, clinical and microbiological data, focusing on predisposing factors, clinical outcomes and 1-year mortality.

**Results:**

Of 1093 patients with probable or definite IE, 19 patients (median age 69 years) had definite GNB-IE, with an increasing incidence throughout the study period. Median age-adjusted Charlson Comorbidity Index score was 4 points. Prosthetic valve IE (PVIE) was present in 7/19 (37%) patients. Nosocomial acquisition occurred in 8/19 (42%) patients. *Escherichia coli* and *Klebsiella pneumoniae* were the most common pathogens. Beta-lactam (BL) based combination therapy was applied in 12/19 (63%) patients (58% BL + fluoroquinolone, 42% BL + aminoglycoside). Cardiac surgery was required in 8/19 (42%) patients (PVIE 71%, native valve IE 25%), primarily for embolism prevention and heart failure. Complications occurred in 14/19 (74%) patients. The in-hospital mortality rate was 21% (4/19); the one-year mortality rate was 44% (7/16). One-year mortality did not significantly differ between patients who underwent cardiac surgery and patients managed with anti-infective treatment alone (*p* = 0.633).

**Conclusions:**

GNB-IE affects elderly patients with high comorbidity levels and recent health-care exposure. GNB-IE was associated with high complication rates and high mortality.

**Supplementary Information:**

The online version contains supplementary material available at 10.1007/s15010-024-02392-w.

## Introduction

Infective endocarditis (IE) due to gram-negative bacilli (GNB) not included in the HACEK group (Haemophilus, Aggregatibacter spp., Cardiobacterium, *Eikenella corrodens*, and Kingella spp.) constitutes 2–3% of IE cases [1, 2]. However, *non-HACEK* GNB-IE is increasingly recognized as an emerging infectious disease (ID) entity with elevated morbidity and mortality [1–4]. Recent research indicated an increase in *non-HACEK* GNB-IE cases, with incidences ranging from approximately 1.8% [2] to 3.9% [3, 5]. These observations have prompted a proposal to update the modified Duke Criteria by including certain gram-negative bacilli as a major criterion for IE, particularly in cases involving intracardiac prosthetic material [6].

The main predisposing factors for *non-HACEK* GNB-IE include advanced age, increased comorbidity, recent health-care contact, immunosuppression, central catheter infections and the presence of a prosthetic heart valve [1–3]. International guidelines now recommend a prolonged combination antibiotic therapy (CT) for *non-HACEK* GNB-IEs based on beta-lactam antibiotics (BL) and aminoglycosides, and in some cases with the addition of fluoroquinolones or cotrimoxazole [7]. Early surgical intervention is recommended, particularly in the case of a prosthetic valve IE (PVIE) [7, 8]. The latest European Society of Cardiology (ESC) guidelines reflected the growing research focus on *non-HACEK* GNB-IE by underscoring the importance of managing GNB-IE [7]. However, due to the rarity of *non-HACEK* GNB-IE, diagnosis can be delayed, and evidence on optimal anti-infective treatment and the indication and timing of cardiac surgery is limited [1]. Emerging drug-resistance among GNB strains poses additional challenges in the management of patients with GNB-IE [7].

To the best of our knowledge, no previous studies have investigated the characteristics and outcomes of patients with IE due to *non-HACEK* GNB in the German health-care setting. In this light, the objective of this retrospective descriptive study was to fill up the evidence gap by providing a detailed description of the predisposing factors, clinical features, management and outcomes of patients diagnosed with *non-HACEK* GNB-IE at a large IE referral center in Germany.

## Methods

We conducted a retrospective descriptive single-center study at Charité – Berlin University Hospital, a tertiary care hospital and referral center for IE patients for conservative management as well as surgical therapy.

We applied the following screening methods to assess study eligibility: first, we identified patients with IE due to *non-HACEK* GNB-IE who were consulted by an ID specialist between 2015 and 2021. In addition, through a review of a pseudonymized extract derived from electronic medical records, we identified patients who were diagnosed with the ICD-10 case codes I33 (“endocarditis”) and B96 (“other specified bacterial agents as the cause of diseases classified to other chapters”, meaning gram-negative bacteria), who were treated in the departments of ID, cardiology or cardio-surgery between 2015 and 2021. The eligibility of the identified patients for the study was then confirmed by a review of the patients’ electronic medical records. We included adult patients who met the criteria for definite *non-HACEK* GNB-IE based on the 2015 ESC diagnostic criteria for IE [9], and who were hospitalized at Charité Hospital between 2015 and 2021. Patients were excluded from the study if the diagnosis of IE was discarded, if blood cultures showed no growth, gram-positive growth, or polymicrobial growth; or if *HACEK* organisms or fungi were identified in blood cultures.

Epidemiological and clinical data were collected retrospectively from patients’ electronic medical records. Nosocomial IE was defined as IE that developed in a patient who had been hospitalized for more than 48 h prior to the onset of signs or symptoms consistent with IE or an infection in general. Comorbidities were assessed with the age-adjusted Charlson Comorbidity Index (ACCI) [10].

Based on previous literature, we considered the following predisposing factors for *non-HACEK* GNB-IE: the presence of a prosthetic valve (PV), defined as the presence of a biological or mechanical prosthetic heart valve; the presence of central intravascular catheters (CVCs); and immunosuppression.

Following the 2015 ESC guidelines for the management of IE, we defined combination antibiotic therapy (CT) as the use of a beta-lactam (BL) in combination with an aminoglycoside or fluoroquinolone [9]. Monotherapy was defined as the use of a single BL antibiotic agent. Treatment regimens that did not include any BLs were categorized as “other”.

The indications for cardiac surgery, including embolism prevention, uncontrolled infection and heart failure, were considered in line with ESC guidelines. Uncontrolled infection was defined as persistent positive blood cultures for more than seven days despite adequate antimicrobial therapy, or locally uncontrolled infections such as cardiac abscesses, pseudoaneurysms, fistulae or enlarging vegetations. Heart failure included dyspnea (NYHA ≥ III), refractory pulmonary edema or cardiogenic shock due to severe heart valve regurgitation or stenosis [9].

Any complication that occurred during the hospital stay was analyzed. Infectious complications included septic shock, persistent bacteremia, secondary infectious foci and sepsis-associated encephalopathy. Thromboembolic or septic embolic events were categorized as embolic events. Cardiac complications included new valvular lesions (regurgitation and/or stenosis ≥ II°) and perivalvular infection. Organ failure included heart failure (as previously defined) and any documented dysfunction or impairment of one or more organs resulting from the infection. In-hospital mortality and 1-year mortality were defined as death from any cause during the hospital stay and at one year after discharge, respectively.

We conducted a retrospective analysis applying descriptive statistics. Continuous variables were regarded as nonnormally distributed and are presented as medians and 25th and 75th interquartile ranges (IQRs). Frequencies (n) and proportions (%) are provided for categorical variables. Quantitative variables were compared using the Mann-Whitney U test. Comparisons of categorical variables were made by either chi-square test or Fisher’s exact test, if the frequency in one group was less than five.

Group comparisons were conducted among predefined subgroups to compare epidemiological and clinical data, as well as management and outcome, between patients with PVIE and patients with native valve IE (NVIE). Additionally, we compared mortality rates between patients who required cardiac surgery in addition to anti-infective therapy, and those managed with anti-infective therapy alone.

For all tests, a *p* value of ≤ 0.05 was considered to indicate statistical significance. Statistical analysis was performed using JMP^®^ Pro 17.1.0 (copyright © 2022–2023 JMP Statistical Discovery LLC. All Rights Reserved.)

The study was performed in accordance with the principles of the Declaration of Helsinki. The need for informed consent was waived in line with the applicable local law (§ 25 LKHG Bln), as data and samples were analyzed retrospectively, and the study was noninterventional in nature.

## Results

**Fig. 1:**
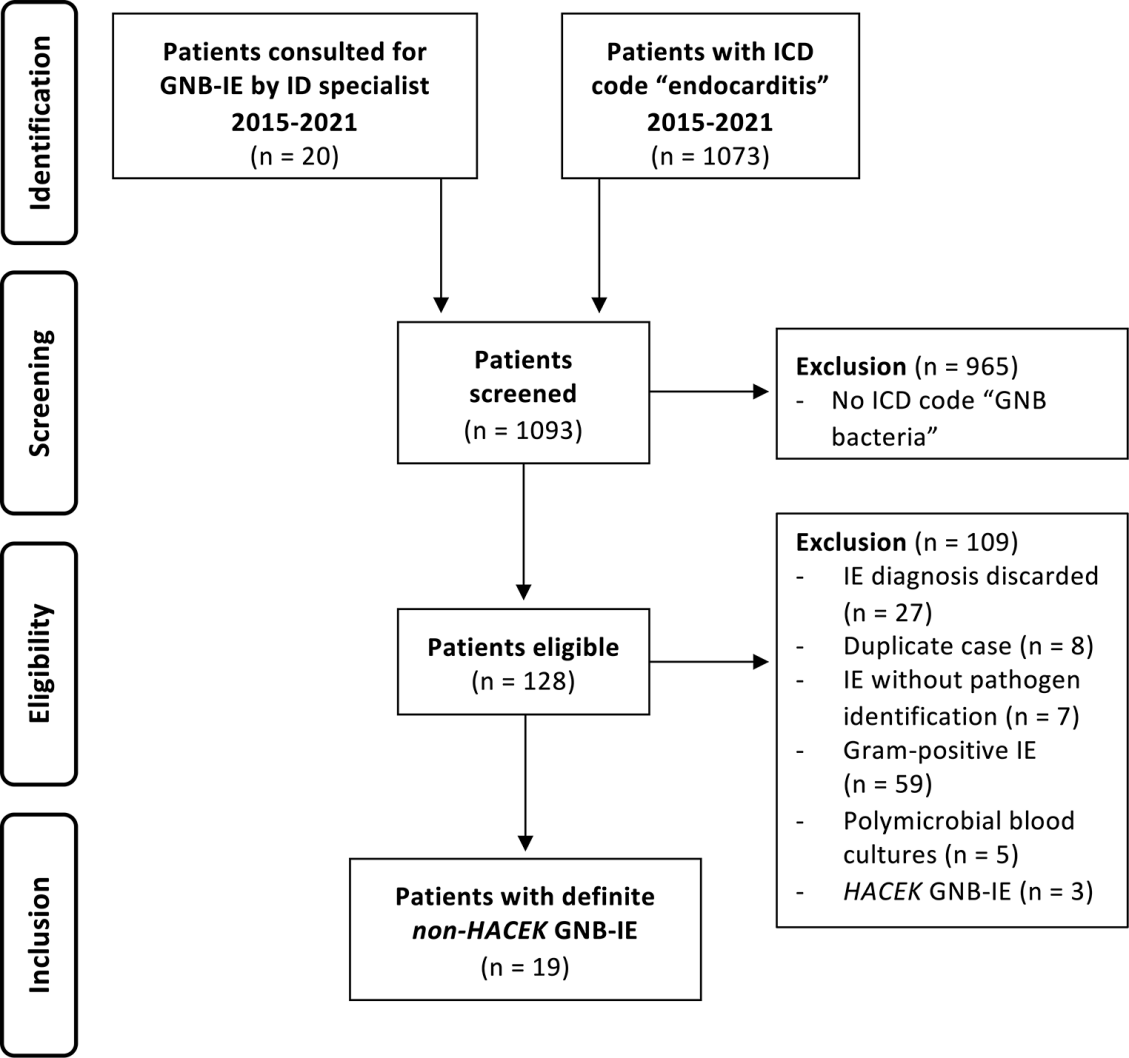
Screening and inclusion process

Of the 1093 patients treated at Charité between 2015 and 2021 who were screened for eligibility, 19 had definite *non-HACEK* GNB-IE. Figure 1 shows a summary of the patient screening and inclusion process.

### Epidemiology, baseline characteristics, echocardiography, microbiology

Between 2015 and 2021, 19 patients with definite *non-HACEK* GNB-IE were diagnosed and treated at our hospital. Throughout the study period, the incidence of *non-HACEK* GNB-IE increased; a single case of *non-HACEK* GNB-IE was observed in 2015, followed by three cases in 2018, four cases in both 2019 and 2020, and seven cases in 2021. For three patients, information regarding the endpoint of mortality at one year was unavailable.

Table 1 shows the participants’ baseline characteristics and predisposing factors. The median age at admission was 69 years (IQR 53–76), and 13 participants (13/19, 68%) were male. The median age-adjusted Charlson Comorbidity Index was 4 points (IQR 2–6). Three participants (3/19, 15%) had a history of previous IE. Vegetations were primarily found on the aortic valve (13/19, 68%) and the mitral valve (5/19, 26%). In one participant, both the aortic and mitral valves were affected. There were no cases of right-sided GNB-IE.

Eight patients (8/19, 42%) met the criteria for nosocomial IE. Predisposing factors were identified in 68% (13/19) of the patients, among whom 54% (7/13) had a prosthetic heart valve, 46% (6/13) had a central venous catheter and 31% (4/13) were immunosuppressed. None of our participants reported intravenous drug use.

**Table 1:** Baseline characteristics

Variable	Frequency *n*/*n* (%), median (IQR)	NVIE *n* = 12	PVIE *n* = 7	*p* value
Male	13/19 (68)	8 (67)	5 (71)	1.0^2^
Age	69 (53–76)	68 (53–75)	75 (53–84)	0.498^1^
ACCI	4 (2–6)	4 (1.25–6.5)	3 (3–6)	0.660^1^
History of IE	3/19 (15)	1 (8)	2 (29)	0.243^2^
Involved valve*				
Aortic valve	14/19 (74)	9 (75)	5 (71)	
Mitral valve	6/19 (32)	4 (33)	2 (29)	
Additional pathological, molecular or microbiological confirmation of intraoperative specimen	6/19 (32)	2 (17)	4 (57)	
Nosocomial IE	8/19 (42)	4 (33)	4 (57)	0.377^2^
Predisposing factors*	13/19 (68)	6 (50)	7 (100)	
PV	7/13 (54)	0 (0)	7 (100)	
CVC	6/13 (46)	4 (33)	2 (29)	
Immunosuppression	4/13 (31)	3 (25)	1 (14)	
Intracardiac electronic device	2/13 (15)	0 (0)	2 (28)	
Possible source of infection*	16/19 (84)	11 (92)	5 (71)	0.532^2^
Urinary	8/16 (50)	6 (50)	2 (29)	
CVC	5/16 (31)	4 (33)	1 (14)	
Gastrointestinal	4/16 (25)	2 (17)	2 (29)	
Other: pulmonary, orodental, soft tissue	3/16 (19)	3 (25)	0 (0)	
Primary bloodstream infection	3/19 (16)	1 (8)	2 (29)	

ACCI: age-adjusted Charlson Comorbidity Index, IE: infective endocarditis, PV: prosthetic valve, CVC: central intravascular catheters. * ≥1 possible, *p* values refer to ^1^the Mann-Whitney-U test, and ^2^chi-square test or Fisher-exact test (if *n* ≤ 5)

**Fig. 2:**
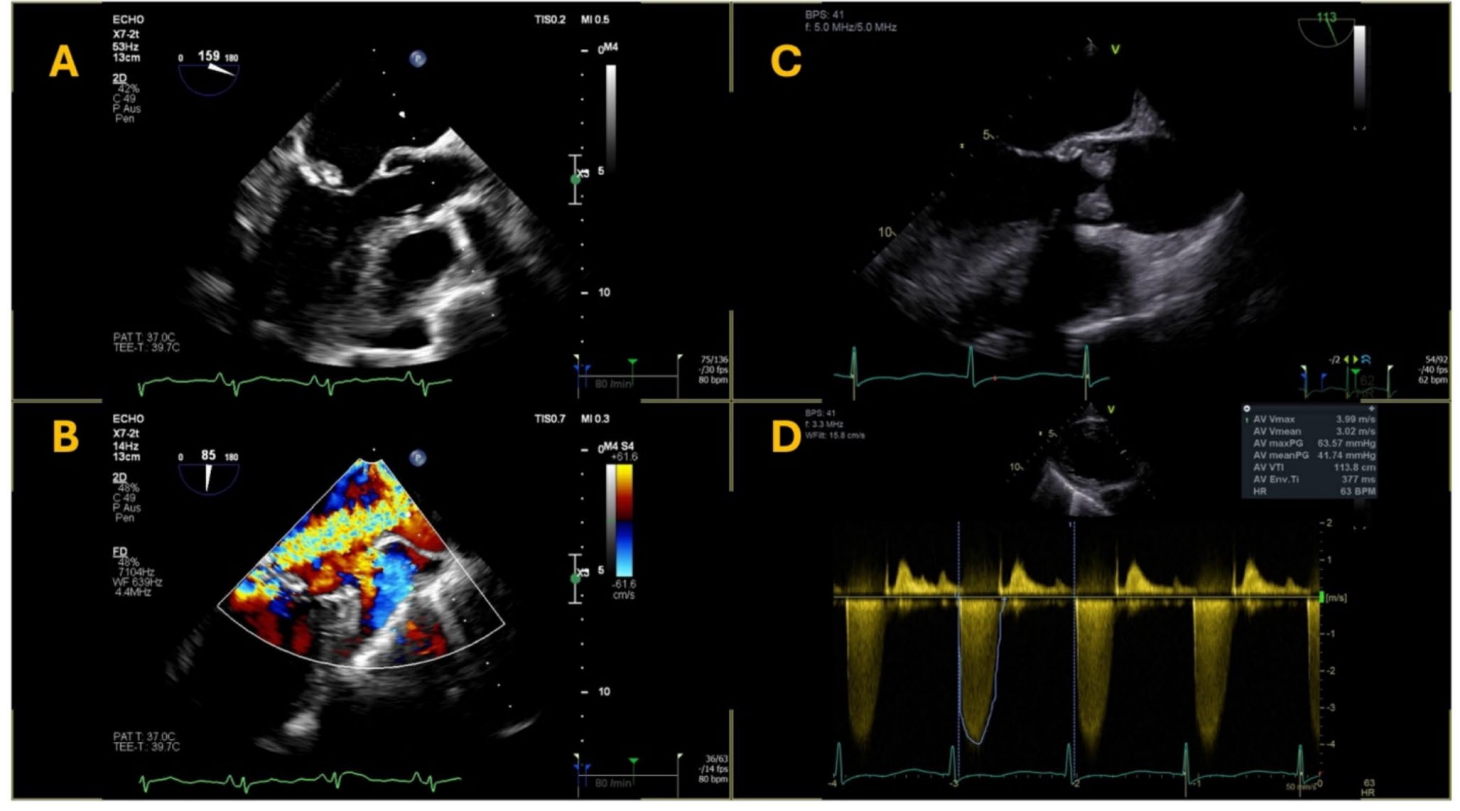
Transesophageal echocardiography findings of two PVIE patients from the analyzed cohort. One patient with native mitral valve endocarditis with a large vegetation on the posterior mitral valve leaflet (Panel **A**) and severe mitral regurgitation. (**B**) The other patient with prosthetic aortic valve endoplastitis (**C**) and severe valvular stenosis caused by large vegetations (**D**)

The PVIE cohort included seven patients with prosthetic valve endoplastitis. All of them were operated via conventional surgery: five with a biological aortic valve prosthesis, one with a biological mitral valve prosthesis, and one with a mechanical mitral valve prosthesis. Endoplastitis occurred within the first two months after surgery in three patients. In the remaining four patients, it occurred 1, 3, 5, or 14 years after surgery. The initial indications for surgery were severe stenosis of the native valve in five patients (73–87 years old), and endocarditis in two patients (32 and 53 years old). One patient diagnosed with endoplastitis of their biological mitral valve had a second prosthetic valve; this biological aortic valve had been implanted in a separate procedure using transcatheter intervention and was not affected by endoplastitis. Figure 2 shows the transesophageal echocardiography findings of two patients with PVIE.

The majority of patients (16/19, 84%) had one or multiple possible sources of infection. Urinary tract infections were the most common possible source of infection (8/16, 50%), followed by central venous catheter infections (5/16, 31%) and gastrointestinal infections (4/16, 25%). Two participants (2/16, 13%) presented with orodental infections, two participants (2/16, 13%) had a pulmonary source of infection, and one participant (1/16, 6%) had a soft tissue infection at admission. In the remaining three patients, no source of infection was identified, and IE was thus categorized as primary blood stream infection.

All participants in the study exhibited positive blood cultures. The most commonly isolated pathogens from blood cultures were *Enterobacterales* (14/19, 74%), including *Escherichia coli* (5/19, 26%), *Klebsiella pneumoniae* (5/19, 26%), *Proteus mirabilis* (3/19, 17%) and *Enterobacter cloacae* (1/19, 5%). In six patients (6/19, 32%), the causative microorganism was additionally confirmed by molecular or pathological analysis of intraoperative specimens or by microbiological findings from a valve culture. Only one patient presented with a multidrug-resistant pathogen, namely, an Oxa-48-carbapenemase-producing *Klebsiella pneumoniae.* A detailed list of all pathogens can be found in Table S3 in the Supplement.

### Treatment, clinical course, outcome

Each patient received a consultation from an ID specialist who recommended parenteral antibiotic treatment in accordance with the guideline recommendations. The recommended treatment duration was six weeks, either following the first negative blood culture or after cardiac surgery, if pathogens were cultured from surgical material. For one patient, parenteral antibiotic therapy was converted to an oral regimen with an antibiotic from the same class as the initial parenteral treatment.

The treatment, clinical course and outcome data are summarized in Table 2. Among the patients who received the recommended combination antibiotic therapy (12/19, 63%), 7/12 (58%) received a BL antibiotic with a fluoroquinolone, and 5/12 (42%) were treated with a BL and an aminoglycoside. Six patients (6/19, 32%) received BL monotherapy. One patient (1/19, 5%) received fluoroquinolone monotherapy. A detailed overview of the causative pathogens, the applied antibiotic therapy and the minimum inhibitory concentrations (MICs) from patients’ resistograms are shown in the supplemental material (Supplement: Table 3).

In-hospital complications occurred in 14/19 (74%) patients, and 8/19 (42%) patients developed two or more complications during their stay. Most patients developed cardiac complications (8/14, 57%), such as cardiac abscesses or new valvular lesions, followed by embolic events (6/14, 43%) and infectious complications (6/14, 43%), such as septic shock, spondylodiscitis or septic encephalopathy. Organ failure occurred in 8/14 (57%) patients.

Cardiac surgery was performed in 8/19 (42%) patients. The majority of patients who underwent cardiac surgery had PVIE (5/8, 63%), and most patients had more than one indication for cardiac surgery. The most common indication for cardiac surgery was embolism prevention in 6/8 (75%) patients, followed by heart failure (5/8, 63%) and uncontrolled infection (3/8, 38%). Nine patients (9/19, 53%) had no indication for cardiac surgery and did not receive surgical intervention, while in two patients (2/19, 12%) surgery, although formally indicated, was not performed due to a prohibitive operative risk. Of these two patients, one was lost to follow-up after returning to their home country, and the other died within 60 days post-discharge.

The in-hospital mortality rate was 21% (4/19), and the most common cause of death was multiorgan failure (3/4, 75%). The overall one-year mortality was 44% (7/16).

Group comparisons between patients with PVIE and patients with native-valve IE (NVIE) revealed no statistically significant differences in clinical outcomes, complication rates, cardiac surgery rates, in-hospital mortality or 1-year mortality (Table 2).

Similarly, we observed no statistically significant differences in mortality rates between patients who underwent cardiac surgery and those who received anti-infective treatment alone. This held true for both in-hospital mortality (25% vs. 18%, *p* = 1.0), as well as 1-year mortality (25% vs. 45%, *p* = 0.633).

**Table 2:** Treatment, clinical course and outcome

Variable	Frequency *n*/*n* (%)	NVIE *n* = 12	PVIE *n* = 7	*p* value
Treatment				
BL monotherapy	6/19 (32)	5 (42)	1 (14)	
CT	12/19 (63)	6 (50)	6 (86)	0.173
BL + fluoroquinolone	7/12 (58)	2/6 (33)	5/6 (83)	
BL + aminoglycoside	5/12 (42)	4/6 (67)	1/6 (17)	
Other	1/19 (5)			
Complications*	14/19 (74)	8 (67)	6 (86)	0.603
Cardiac complications*	8/14 (57)	4 (33)	4 (57)	
Cardiac abscess	6/8 (75)	3 (25)	3 (43)	
New valvular lesions	6/8 (75)	4 (33)	2 (29)	
Embolic events	6/14 (43)	3 (25)	3 (43)	
Infectious complications*	6/14 (43)	4 (33)	2 (29)	
Septic shock	2/6 (33)	1/4 (25)	1/2 (50)	
Spondylodiscitis	2/6 (33)	2/4 (50)	0/2 (0)	
Other	4/6 (67)	3/4 (75)	1/2 (50)	
Organ failure	8/14 (57)	6 (50)	2 (29)	
Cardiac surgery	8/19 (42)	3 (25)	5 (71)	0.074
Indications for cardiac surgery*				
Embolism prevention	6/8 (75)	2/3 (67)	4/5 (80)	
Heart failure	5/8 (63)	2/3 (67)	3/5 (60)	
Uncontrolled infection	3/8 (38)	1/3 (33)	2/5 (40)	
In-hospital mortality	4/19 (21)	3 (16)	1 (14)	1.000
Multiorgan failure	3/4 (75)	2/3 (67)	1/1 (100)	
Hypoxemia	1/4 (25)	1/3 (33)	0/1 (0)	
1-year all-cause mortality	7/16 (44)	5/9 (56)	2/7 (29)	0.358
No data at 1-year follow-up	3/19 (16)			

NVIE: native valve infective endocarditis, PVIE: prosthetic valve infective endocarditis, BL: beta-lactam, CT: combination therapy. * ≥1 possible, *p* values refer to chi-square test or Fisher-exact test (if *n* ≤ 5)

## Discussion

To the best of our knowledge, this is the first study to describe the characteristics and outcomes of patients with *non-HACEK* GNB-IE in Germany.

The principal findings of our study highlight the significant clinical severity and high mortality observed among patients with *non-HACEK* GNB-IE. One-fifth of patients died during hospitalization, and nearly one half died within the first year after discharge. In recent multicenter studies, comparable morality rates in IE due to *non-HACEK* GNB-IE were observed, ranging from 14% to 47% [1–3, 11]. Elevated mortality in patients with *non-HACEK* GNB-IE has been associated with advanced age, PVIE, and not undergoing cardiac surgery [1, 11, 12]. However, the decision to forgo surgery can be multifactorial and complex and is frequently driven by the high risk of intraoperative mortality due to patient morbidity.

The high mortality rate observed in our study may be attributable to the distinctive risk profile of advanced age and increased comorbidity characteristic of patients with *non-HACEK* GNB-IE and may have been compounded by the high rate of complications [1–3, 5]. In line with recent literature, patients with *non-HACEK* GNB-IE in our study were older than a representative cohort of patients in the International Collaboration on Infective Endocarditis study who had IE due to other microorganisms [2]. We observed high comorbidity levels measured by the ACCI [1]; however, comparisons with a representative group of patients with IE due to other causes were limited by heterogeneous measurements. Despite the male predominance, the proportion of male patients with *non-HACEK* GNB-IE in our cohort was comparable to that of patients with IE due to other microorganisms [2].

In our study, the majority of patients received a BL-based CT, as it is currently recommended by international guidelines. The current recommendations for *non-HACEK* GNB-IE anti-infective therapy are primarily based on expert opinion or past guidelines, advocating for a combination of a BL with an aminoglycoside, sometimes with additional fluoroquinolones or cotrimoxazole [7, 8, 13]. However, previous cohort studies do not provide strong evidence on the superiority of a CT over a BL-based monotherapy for *non-HACEK* GNB-IE, and both treatment approaches demonstrate similar mortality rates [1, 2, 14, 15]. One recent retrospective cohort study reported increased readmission rates in patients who received CT, which may be attributed to increased toxicity associated with the administration of non-BL antibiotics [11]. Given the potential toxicity of combination agents such as aminoglycosides and the lack of evidence of the superiority of CT over BL monotherapy, the routine use of CT should be questioned.

The rates of cardiac surgery in our study were consistent with those reported in prior studies [1, 2, 16–18]. In accordance with current guideline recommendations, the majority of patients diagnosed with PVIE underwent cardiac surgery, whereas only one-quarter of patients with NVIE required surgical intervention. One-year post-discharge mortality did not significantly differ between patients who underwent cardiac surgery and those managed with anti-infective treatment alone. Surgery is recognized as a key pillar in the management of IE due to *non-HACEK* GNB, especially in the case of prosthetic valve IE [7]. This intervention is considered crucial for pathogen eradication and for mitigating the risk of complications and antibacterial resistance [9, 19]. A recent study including 104 patients with *non-HACEK* GNB-IE revealed that not performing surgery despite indications was associated with a higher mortality rate [1]. However, in line with our results, several studies reported no difference in outcomes in patients with *non-HACEK* GNB-IE who received cardiac surgery compared to patients who were managed medically [1, 2, 15, 18]. It could be concluded that surgical intervention for patients with more severe IE might achieve outcomes comparable to those of anti-infective therapy in patients with less severe *non-HACEK* GNB-IE.

It is essential to acknowledge the limitations imposed by the small study cohort, the diverse microorganism profiles and the limited duration of patient follow-up, preventing definitive conclusions regarding the indications for cardiac surgery. Larger prospective cohort studies are needed to determine the role of surgical intervention in the management of *non-HACEK* GNB-IE, ideally in relation to the causative GNB. The interdisciplinary complexity of managing IE patients has been recognized, and a recent systematic review and meta-analysis demonstrated the positive impact of dedicated multidisciplinary IE teams on surgical and patient outcomes, including lower short-term mortality rates [20]. During the timeframe of the current study, IE cases were discussed regularly by an interdisciplinary IE team. Given the severity and high mortality of the disease, there is a critical need for the multidisciplinary IE team to be involved in the management of IE due to *non-HACEK* GNB-IE.

Consistent with previous studies, we observed a high rate of nosocomial IE. Accordingly, most patients exhibited a combination of healthcare-associated risk factors for GNB-IE, such as prosthetic heart valves, CVCs or intracardiac devices [1, 2, 15]. An overall increase in valve replacement surgeries among elderly patients has been reported, resulting in a greater proportion of PVIE within the GNB-IE patient population [12, 21, 22]. A recent study reported that one to six percent of all patients with heart valve prostheses were diagnosed with PVIE. Furthermore, more than 20% of all cases of IE were classified as PVIE [4]. These observations highlight the importance of previous health-care contact and related invasive medical procedures as major risk factors for the development of *non-HACEK* GNB-IE. In contrast to previous studies that have linked *non-HACEK* GNB-IE to intravenous drug use [11, 15], none of our patients reported such behavior.

In line with recent studies, IE due to *Enterobacterales* was more frequent than IE due to nonfermenting GNB, with *Escherichia coli* and *Klebsiella pneumoniae* being the most prevalent pathogens [1, 3, 23]. Global reports have shown a rise in GNB-IE, which may be attributed to the growing proportion of GNB blood stream infections, particularly *Enterobacterales* [24], as well as to an increase in age and comorbidity levels in the hospitalized population. In line with previous studies, we found that urinary tract infections were the most common presumed source of infection for *non-HACEK* GNB-IE, highlighting their importance as an entry point for GNB [1–3, 23].

Our study has several limitations. First, the retrospective nature of the study design may have introduced inherent biases due to the incompleteness and limited reliability of the electronic medical records as the main data source. Second, the study was conducted as a single-center study at one university hospital, and due to the low incidence of the disease, the sample size was small. Third, our university hospital may have served as a referral center for complicated IE cases with an indication for cardiac surgery, which may have led to selection bias with an overrepresentation of patients with complicated clinical trajectories. Last, the various statistical comparisons within a limited study sample size could have led to decreased precision in estimations and an increased likelihood of type I errors.

In conclusion, GNB-IE affects an elderly patient cohort with recent health-care contact and high comorbidity levels. Although recommended combination antibiotic therapy was applied in a majority of patients, we found high rates of complications, frequent indications for cardiac surgery and high mortality rates. In summary, in the case of persistent or recurrent bacteremia due to *non-HACEK* organisms, IE should be suspected, especially in elderly patients with health-care-associated risk factors. Case consultation by an ID specialist and the involvement of a multidisciplinary IE team are especially crucial for the successful management of patients with infective endocarditis caused by *non-HACEK* GNB.

## Electronic supplementary material

Below is the link to the electronic supplementary material.

Supplementary Material 1

## Data Availability

No datasets were generated or analysed during the current study.

## Declarations

### Use of artificial intelligence tools

AI was used to correct spelling and grammar.
