# Supplementary material for: Non-HACEK gram-negative bacilli infective endocarditis: data from a retrospective German cohort study
Source: Infection. 2024 Sep 19;53(1):405–13. doi: 10.1007/s15010-024-02392-w (PMC11825580; doi:10.1007/s15010-024-02392-w)
Supplement: Supplementary file 1 — Supplementary Material 1 [file 15010_2024_2392_MOESM1_ESM.docx]

Supplement

**Table** **3 – Pathogens, anti-infective treatment, MIC**

| Pathogen | Affected valve | Antimicrobial agent class | Antimicrobial agent | MIC (mg/L) | Dosing | Surgery | Outcome |
| --- | --- | --- | --- | --- | --- | --- | --- |
| *Achromobacter sp.* | Native AV | β-lactam | Piperacillin/tazobactam | S 1.5 | 4.5g 3x/d | No | LTFU |
| *Enterobacter cloacae* | Prosthetic AV | β-lactam | Meropenem | S ≤ 0.25 | 2g 3x/d | Yes | Survival |
|  |  | Aminoglycoside | Gentamicin | I ≤ 1 | 3mg/kg |  |  |
| *Escherichia coli* | Native AV | β-lactam | Ceftriaxone | No MIC | 2g 1x/d | No | Survival |
|  |  | Fluoroquinolone | Ciprofloxacin | No MIC | 400mg 2x/d |  |  |
| *Escherichia coli* | Native MV | Fluoroquinolone | Ciprofloxacin | S ≤ 0.25 | 400mg 2x/d | Yes | Survival |
| *Escherichia coli* | Prosthetic AV | β-lactam | Ceftriaxone | S (Cefotaxime ≤ 1) | 2g 1x/d | Yes | Survival |
|  |  | Fluoroquinolone | Ciprofloxacin | S 0.25 | 400mg 2x/d |  |  |
| *Escherichia coli* | Native AV | β-lactam | Ceftriaxone | S (Cefotaxime ≤ 1) | 2g 1x/d | No | Ceased |
|  |  | Aminoglycoside | Gentamicin | S ≤ 1 | 3mg/kg |  |  |
| *Escherichia coli* | Native AV | β-lactam | Ceftriaxone | Not available | 2g 1x/d | No | LTFU |
|  |  | Aminoglycoside | Gentamicin | Not available | 3mg/kg |  |  |
| *K. pneumoniae* | Prosthetic AV | β-lactam | Ceftriaxone | S (Cefotaxime ≤ 1) | 2g 1x/d | Yes | Ceased |
| *K. pneumoniae* | Native MV | β-lactam | Ceftriaxone | S (Cefotaxime ≤ 1) | 2g 1x/d | No | Ceased |
|  |  | Aminoglycoside | Gentamicin | S ≤ 1 | 3mg/kg |  |  |
| *K. pneumoniae,* ESBL positive | Native AV | β-lactam | Meropenem | S ≤ 0.25 | 1g 3x/d | No | LTFU |
|  |  | Aminoglycoside | Gentamicin | S ≤ 1 | 3mg/kg 1x/d |  |  |
| *K. pneumoniae* | Native AV | β-lactam | Ceftriaxone | S (Cefotaxime ≤ 1) | 2g 1x/d | No | Survival |
| *K. pneumoniae,* Oxa-48 positive | Native AV, native MV | β-lactam | Ceftazidime/avibactam | S ≤ 1 | 2.5g 3x/d | No | Ceased |
| *Moraxella lacunata* | Native AV | β-lactam | Meropenem | No MIC | 2g 3x/d | Yes | Survival |
| *Proteus mirabilis* | Prosthetic AV | β-lactam | Cefotaxime, (amoxicillin) | S ≤ 1,  (S ≤ 0.25) | 2g 3x/d | No | Ceased |
|  |  | Fluoroquinolone | Ciprofloxacin | S ≤ 0.25 mg/L | 500mg 2x/d orally |  |  |
| *Proteus mirabilis* | Native MV | β-lactam | Meropenem,  (Cefotaxime) | S ≤ 0.25,  (S ≤ 1) | 2g 3x/d | Yes | Ceased |
|  |  | Fluoroquinolone | Ciprofloxacin | S ≤ 0.25 | 400mg 2x/d |  |  |
| *Proteus mirabilis* | Prosthetic MV | β-lactam | Ampicillin/ sulbactam | S ≤ 2 | 3g 3x/d | Yes | Survival |
|  |  | Fluoroquinolone | Ciprofloxacin | S ≤ 0.25 | 400mg 2x/d |  |  |
| *Pseudomonas aeruginosa* | Native AV | β-lactam | Meropenem | S 0.5 | 2g 3x/d | No | Survival |
| *Pseudomonas aeruginosa* | Prosthetic MV | β-lactam | Ceftazidime | I 4 | 2g 3x/d | Yes | Survival |
|  |  | Fluoroquinolone | Ciprofloxacin | I ≤ 0.25 | 400mg 2x/d |  |  |
| *Raoultella ornithinolytica* | Native AV | β-lactam | Ceftriaxone,  (→ Ceftazidime) | S,  (S ≤ 0.25) | 2g 1x/d,  (2g 3x/d) | No | LTFU |
|  |  | Fluoroquinolone | Ciprofloxacin | S ≤ 1 | 400mg 2x/d |  |  |
| AV: aortic valve, MV: mitral valve. MIC: Minimal inhibitory concentration with breakpoints S (sensitive), I (increased exposure), R (resistant) according to EUCAST. LTFU: Lost to follow-up. Medication was administered intravenously unless specified; duration of therapy was six weeks. | | | | | | | |
